# Obstructive Sleep Apnea Following Secondary Velopharyngeal Insufficiency in Children with Non-Syndromic Cleft Palate: A Systematic Review

**DOI:** 10.3390/cmtr18010006

**Published:** 2025-01-03

**Authors:** Milton Chin, Mona Haj, Sarah L. Versnel, Henriette H. W. de Gier, Eppo B. Wolvius

**Affiliations:** 1 Department of Maxillofacial Surgery, Erasmus MC–Sophia Children’s Hospital, University Medical Centre Rotterdam, 3015 GD Rotterdam, The Netherlands; 2 Department of Plastic and Reconstructive Surgery, Erasmus MC–Sophia Children’s Hospital, University Medical Centre Rotterdam, 3015 GD Rotterdam, The Netherlands; 3 Department of Otorhinolaryngology and Head and Neck Surgery, Erasmus MC–Sophia Children’s Hospital, University Medical Centre Rotterdam, 3015 GD Rotterdam, The Netherlands

**Keywords:** cleft palate, pharynx, velopharyngeal insufficiency, obstructive sleep apnea, sleep apnea, obstructive*/etiology

## Abstract

Study design: Systematic review. Objective: Obstructive sleep apnea (OSA) is a possible complication following secondary velopharyngeal insufficiency surgery in patients with repaired cleft palate. Various surgical techniques are used to treat secondary velopharyngeal insufficiency after cleft palate repair, but the optimal procedure remains debatable. This review provides an overview of the incidence of airway obstructive outcomes related to different surgical modalities. Methods: A systematic search was performed on the 1st of February following the PRISMA guidelines and registered on PROSPERO (CRD42022299715). The following databases were reviewed: Medline, EMBASE, Web of Science, Google Scholar, and the Cochrane Library databases. Studies that included data on the occurrence of OSA following velopharyngeal surgery in children with a repaired non-syndromic cleft palate were included. Non-English articles and studies that included syndromic cleft palate patients were excluded. Results: Twenty-eight articles met the inclusion criteria. The surgical procedures are classified into three groups: pharyngeal flap procedure (PF), sphincter pharyngoplasty (SP), and palatal muscle repositioning (PMR). Incidence of post-operative OSA and symptoms of OSA were lowest after PMR compared to SP and PF (3%; 34%; 29%, respectively). Pharyngeal flap procedures resulted in the best speech outcomes. Conclusions: PMR results in fewer postoperative complications in terms of OSA and achieves a satisfactory reduction in hypernasal speech. PF procedure carries a higher risk of developing OSA postoperatively but seems to be superior in the reduction in hypernasality.

## 1. Introduction

Velopharyngeal insufficiency (VPI) is a well-known condition in patients with unrepaired cleft palate and is normally treated with the surgical repair of the cleft palate. However, in 30–80% of the cases, the velopharyngeal function remains insufficient due to inadequate structural repair, shortening of velum, or immobility of the velopharyngeal sphincter [1,2,3,4,5]. If velopharyngeal insufficiency persists after primary repair of the palate, the indication for secondary velopharyngeal surgery will be considered after conducting a naso-endoscopy [6]. The surgical procedures to treat VPI can be divided into three categories: palatal muscle repositioning, pharyngoplasty, and posterior wall augmentation [6]. These procedures respectively aim to improve velopharyngeal function by restoring the local anatomy or by creating a minor or significant obturation of the nasopharyngeal port based on pre-operative assessment of the velopharyngeal region. Velopharyngeal competence, as evidenced by speech without nasal air escape and the ability to increase intra-oral pressure during speech production, is established in most patients who undergo secondary procedures for the treatment of VPI [6,7].

Following VPI surgery the anatomical changes of the oro-and nasopharynx combined with the reduction in the pharyngeal airway dimensions may contribute to the increased risk of developing obstructive airway symptoms or lead to OSA [8,9]. Polysomnography (PSG) is the gold standard for objective measurement of the sleep pattern and respiratory parameters for diagnosing OSA. Apnea-Hypopnea Index (AHI), Oxygen Desaturation Index (ODI) and Respiratory Disturbance Index (RDI) are well established parameters to objectify the frequency of events during PSG. OSA in adults is diagnosed if five or more respiratory events per hour are observed [10,11]. The pediatric criteria for diagnosis of OSA include one or more obstructive events in a patient with pre-existing symptoms (snoring, obstructed/labored breathing, or daytime consequences) [12].

The effects of secondary pharyngeal surgery on speech outcomes are elaborately reported in the literature [6]. However, the airway complications following various techniques remain poorly investigated. Recently, Kurnik et al. evaluated the occurrence of OSA after VPI re-repair in a meta-analysis [13]. The authors mainly focused on palatal re-repair and included a small number of studies for the alternative surgical methods.

Considering the significant implications of OSA on mental health, cardiovascular status, and quality of life, the void in the literature regarding its incidence following VPI re-repair is inconceivable. This paper aims to determine the incidence of post-operative airway obstructive outcomes (short- and long-term) following different surgical treatments of VPI in patients with non-syndromic cleft palate who have previously undergone palatal closure. Additionally, short- and long-term speech outcomes that are reported in the included studies following various surgical techniques will be evaluated.

## 2. Materials and Methods

PICO question: What is the incidence of obstructive sleep apnea following surgical treatment of VPI in non-syndromic patients with a repaired cleft palate?

P: Non-syndromic patients with a cleft palate who have previously undergone palatal closure.

I: Pharyngeal Flap surgery, Sphincter Pharyngoplasty surgery, Palatal Muscle Repositioning surgery

C: -

O: Incidence of post-operative OSA (symptoms/diagnosis) at short-term (<1 month) and long-term (>1 month) follow-up.

### 2.1. Search

Studies were identified by searches of the following databases: Medline, EMBASE, Web of Science, Google Scholar, and the Cochrane Library databases using the following search terms: ”velopharyngeal Insufficiency”, “pharyngoplasty”, “apnea”, “polysomnography”, “cleft palate”. No search restrictions with respect to publication language or dates were applied. References were included based on relevance to the subject of the manuscript, with priority given to reports of prospective randomized trials; if this level of evidence was absent, cohort studies or reviews of such studies were selected for inclusion. The search was performed on 1 February 2024. The search strategy can be found in the Supplementary Digital Content (SDC): Supplementary S1.

### 2.2. Selection

The protocol for the systematic review was registered on PROSPERO (CRD42022299715). Analysis was performed in accordance with the Preferred Reporting Items for Systematic Reviews and Meta-Analyses (PRISMA) [14]. A two-stage screening was conducted using Endnote X8/9 and Excel. Initially, the title and abstract were independently screened and assessed by two reviewers. Studies were accepted into the second stage of screening if they included non-syndromic patients after primary cleft repair and reported obstructive airway outcomes after a surgical intervention to improve speech. Duplicates, non-relevant papers, and papers that did not comply with the inclusion criteria were excluded. During the second stage, the full-text of the remaining articles was thoroughly read and reviewed by two reviewers.

Studies were included if they met the following inclusion criteria: non-syndromic patients with a repaired cleft palate undergoing secondary surgery for VPI; reporting on outcomes of post-operative airway obstruction; pharyngoplasty procedures; palatal muscle repositioning procedures. Studies were excluded if no range of follow-up was mentioned. Consequently, non-English articles, case reports, commentaries, letters, and duplicates were also excluded. Discrepancies between reviewers were resolved by discussion, if this failed another author was addressed to adjudicate for final judgment. To prevent the inclusion of overlapping patient populations, we compared the authorship and publication and recruitment date, source, and location. If ambiguity was present with regards to overlapping patient populations of two studies, the author was contacted to clarify. Recognized overlap was dealt with by the inclusion of the most recent and inclusive follow-up study.

### 2.3. Assessment of Risk of Bias

The reviewers assessed the quality of the studies using the Oxford Centre for Evidence-Based Medicine (2011) level of Evidence guidelines [15] and the Cochrane Collaboration’s tool for assessing the risk of bias [16]. This was performed using the RevMan software version 8.13.0 [17]. Studies were assessed on selection bias, detection bias, outcome reports (OSA and speech outcomes), reporting bias, and attribution bias.

### 2.4. Extraction

Surgical procedures were divided into the following categories: pharyngeal flap (PF); sphincter pharyngoplasty (SP); palatal muscle repositioning (PMR). The number of patients with the diagnosis of OSA (based on AHI, ODI, or RDI) and clinical signs of OSA after surgical VPI correction was defined as the primary outcome variable. Speech was considered as a secondary outcome and was classified into the following groups: competent VPI function; normal resonance, no nasal air emission; normal intra-oral pressure; closed velopharyngeal gap; normal speech intelligibility; normal articulation.

### 2.5. Data Synthesis

Extracted data were processed in Excel and arranged according to the follow-up periods divided into two groups: short- and long-term (≤one month and >one month). This distinction will help to identify temporary airway obstructive symptoms due to post-operative edema.

### 2.6. Pooling Method

The total number of participants and incidence (%) of symptoms of OSA and diagnosis of OSA per study were extracted to Excel. By using the following formula, we calculated the total number of participants with symptoms or diagnosis of OSA for each study:

(1) $$Incidence\;OSA\;\left(\%\right)\times Study\;population=Total\;number\;of\;affected\;participants.$$

The data from the included studies were then divided into groups based on the surgical technique and follow-up period. The incidence per surgical technique was then calculated by the following formula:

(2) $$\frac{Total\;number\;of\;affected\;patients\;following\;a\;specific\;VPI\;repair}{Total\;number\;of\;patients\;that\;have\;undergone\;VPI\;repair}\;\;\;\;\;\;\;\;\;\;\;\;\;=Incidence\;\left(\%\right)\;of\;OSA\;or\;diagnosed\;OSA\;after\;a\;specific\;VPI\;repair$$

A similar calculation was performed with regard to the speech outcome.

These data were processed into figures to provide a visual overview of the various techniques and follow-up periods. The pooling method was approved by the statistics department of the Erasmus MC.

## 3. Results

**Figure 1:**
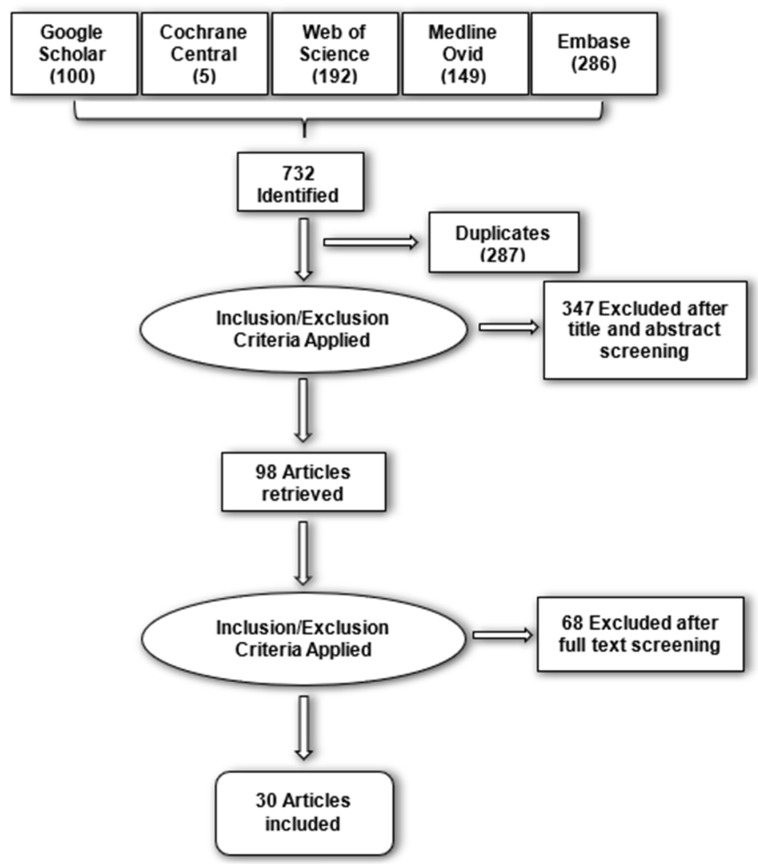
A figure that demonstrates the PRISMA flow diagram.

Initially, a total of 732 papers were identified, of which 98 remained when the duplicates (287) and unrelated papers based on title and abstract 68 were removed. A total of 30 papers were finally eligible for inclusion after a full-text screening. The PRISMA guideline flowchart can be found in Figure 1.

### 3.1. Study Characteristics

This review consisted of 16 prospective and 11 retrospective case series and three randomized controlled trials. The PF procedure, reported in 18 studies, SP reported in 12 studies and PMR reported in 10 studies, were the most common procedures among the surgical modalities.

The majority of patients (66%) in PMR-group underwent a Furlow Z-palatoplasty. In only one study intravelar veloplasty (IVP) was performed. Two studies used the bilateral buccinator myomucosal flap [18,19,20].

The median follow-up was 1 year (Interquartile range (IQR): 2.02 years). The median age at VPI repair was 8.9 years (IQR: 11.33 years) for the PF procedure. The median age for the SP procedure was 14 years (IQR: 7.58 years). For the PMR procedure, the median age was 8 months (IQR: 6.3 years).

A total of 1349 patients were included. Of these, 628 patients underwent PF, 460 patients underwent PMR, and 261 patients underwent SP (Table 1).

In 23 studies a post-operative evaluation of the presence and severity of OSA using PSG was performed. In twelve of these studies, PSG was performed in all patients post-operatively, while in other studies PSG was only performed in patients who presented symptoms of airway obstruction post-operatively. The remaining studies were unclear about the use of PSG or only reported clinical signs of OSA (for example using questionnaires). Twenty-five out of 30 studies reported outcomes on OSA after one one-month period post-operatively. Nine studies reported the presence of clinical symptoms or PSG results pre-operatively.

### 3.2. Primary Outcomes

The symptoms and diagnosis of OSA in the PMR group were reported in 2% and 3% of the studied patients after short-term follow-up. In long-term follow-up, PMR resulted in a lower incidence of OSA (3%) and its symptoms (3%).

**Figure 2:**
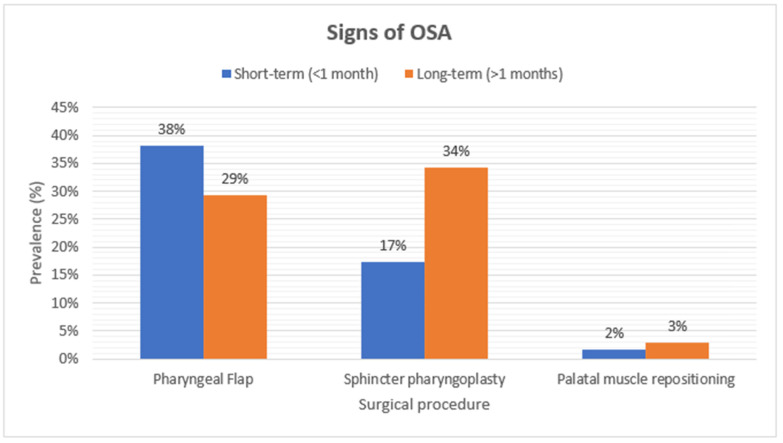
A figure that demonstrates the incidence of signs of OSA of the included studies.

**Figure 3:**
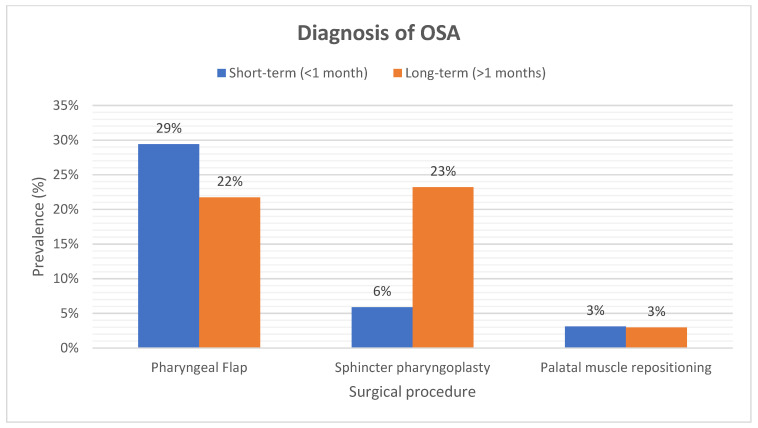
A figure that demonstrates the post-operative diagnosis of OSA after PSG.

Short-term results of PF surgery showed that 38% of the patients had clinical signs of OSA. In the long-term, the incidence of symptoms decreased to 29% (Figure 2). PSG in the long-term revealed OSA in 22% of patients (Figure 3). Studies also reported a higher Apnea Hypopnea Index (AHI) following the PF procedure in comparison to SP and PMR [18,21,22,23].

Seventeen percent of patients who received SP were symptomatic at short-term follow-up. In the long-term, 34% of the patients after SP showed clinical signs of OSA. PSG in the long-term revealed OSA in 23% of patients.

### 3.3. Secondary Outcomes

**Figure 4:**
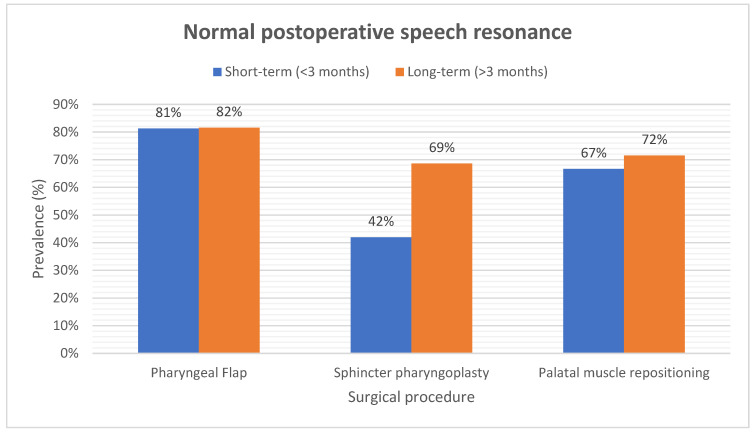
A figure that demonstrates the incidence of a normal postoperative speech resonance in the included studies.

The current review reveals normal resonance in 81% of the study subjects after PF surgery and in 42% after SP procedure at short-term follow-up. Similar results in PF- and SP groups were seen at long-term follow-up (80% and 69% normal resonance, respectively). Sixty-seven percent of the PMR patients achieved a normal resonance at short-term follow-up. This number increased to 72% at long-term follow-up (Figure 4).

In the long-term, a competent velopharyngeal function was found in 82% of the patients that underwent PF surgery. This was 69% and 77% in the SP and PMR groups, respectively (SDC Figure S1).

Results on other speech parameters (e.g., articulation, intra-oral pressure, speech intelligibility, closed velopharyngeal gap) can be found in the SDC (Figures S2–S6).

### 3.4. Quality Assessment

Out of three randomized controlled trials (10%), only one study had a low risk of selection bias (3.3%). The other remaining studies scored level IV on the Oxford Centre of Evidence-Based Medicine (OCEBM) evidence tool and consisted of prospective and retrospective case series. The majority of the studies (90%) carried a high risk of selection bias, because the allocation of treatment was determined by the clinicians. Twenty-four studies (80%) reported OSA based on PSG results. Nineteen studies (63.3%) reported speech outcomes. For the detection bias, six studies (20%) used a blinded speech assessment. The reviewers determined that the lack of blinding is unlikely to affect the assessment of OSA. However, the risk of the speech assessment remains unclear. No high risk of reporting and attribution bias was found in the included studies.

An overview of the risk of bias assessment can be found in Table 2 An additional figure of the risk of bias can be found in the supplementary digital content (SDC Figure S7).

**Table 1:** A table that shows an overview of the characteristics of the included studies.

References (Year)	Study Type	Procedure	Number of Patients (*n*)	Cohort Period	Mean Follow-Up Time (Range)	Mean Age VPI Surgery (Range)	Type of Primary Palatal Repair	Pre-Operative OSA (Clinical or PSG) (%)	Post Operative OSA Symptoms ^a^		Post Operative OSA (Using PSG)		Post-Operative Normal Resonance ^b^ (%)
									Short-Term (%)	Long-Term (%)	Short-Term (%)	Long-Term (%)	
Abdel-Aziz et al. (2008) [24]	Prospective cohort	SP	17	2003–2006	6 months	7 years 2 months	NR	NR	NR	0	-	0	52.9
Abdel-Aziz et al. (2011) [25]	Prospective cohort	PF	22	2006–2008	6 months	9 years 8 months	primary pushback palatoplasty	NR	-	27.3	-	13.6	90.9
		SP	26						-	3.85	-	0	88.4
Abdel-Aziz et al. (2018) [21]	Prospective cohort	PF	9	2015–2017	at least 6 months	7 years 5 months	NR	NR	-	77.8	-	77.8	NR
		SP	18						-	55.6	-	55.6	NR
		PMR	12						-	25	-	25	NR
Boynuyoğun et al. (2023) [26]	Retrospective cohort	PF	72	2020–2021	6 months	10 years and 6 months	Two flap; Furlow; Langenbeck	0	-	6.9	-	6.9	NR
Abyholm et al. (2004) [27]	Randomized controlled trial	PF	22	1993–1996	12 months	3–25 years	NR	0	50	43.8	22.7	21.4	80.8
		SP	21					9.52	14.3	10	15	6.25	75.6
Campos et al. (2016) [28]	Prospective cohort	PF	22	2011–2012	>20 years (after surgery)	48 years 6 months	NR	NR	-	91	-	63.6	NR
Chen et al. (1996) [29]	Retrospective cohort	PMR	30	1988–1993	6 months–4 years	3–26 years	simultaneous primary repair	NR	-	0	-	0	96.7
Denadai et al. (2017) [19]	Prospective cohort	PMR *	53	2010–2015	15 months	>7 years	NR	0	0	0	0	0	71.7
El Anwar et al. (2017) [30]	Prospective cohort	PF	10	2014–2016	10 months (6–22)	6 year 11 months	NR	NR	-	0	-	0	90
Elsherbiny et al. (2020) [20]	Prospective cohort	PMR *	30	2013–2015	at least 6 months	10 years and 9 months (4.5–9 years)	NR	NR	-	-	-	0	25
Emara et al. (2012) [31]	Prospective cohort	PF	26	2007–2010	10–31 months	5–16 years	NR	NR	15.4	-	15.4	-	92.3
Hassib et al. (2005) [32]	Randomized controlled trial	PF	11	2000–2003	10.4 months	3 years 5 months	NR	NR	-	18.2	-	18.2	81.8
		SP	11			3 years 9 months			-	0	-	0	72.7
Hsu et al. (2015) [33]	Retrospective cohort	PMR	13	2005–2014	33.7 months (10 to 95)	10 years and 8 months	double-opposing Z-plasty	NR	-	7.67	-	0	69.2
Junior et al. (2012) [34]	Retrospective cohort	PF	39	2000–2010	5–10 years	19 years 11 months	NR	NR	0	10.3	0	10.3	NR
Liao et al. (2002) [22]	Prospective cohort	PF	38	1996–1999	at least 6 months	children and adults	NR	0	-	94.7	-	92.1	NR
Liao et al. (2003) [35]	Prospective cohort	PMR	10	1997–1998	6 months	5 years 1 months	NR	30	100	50	100	50	NR
Lin et al. (1999) [23]	Retrospective cohort	PF	10	1986–1996	61.0 months (38–96)	1 year 2 months	cleft repair + levator retropositioning	NR	-	NR	-	NR	80
		PMR	14		64.3 months (36–88)	1 year	simultaneous double opposing Z plasty		0	0	0	0	57.1
Liu et al. (2020) [36]	Retrospective cohort	SP + PMR ***	31	2013–2017	6–36 months	15	simultaneous primary repair	NR	19.4	19.4	0	0	51.6
		PMR ***	27			18			0	0	0	0	22.2
Luo et al. (2019) [37]	Retrospective cohort	PF	30	2009–2011	6 months	16	NR	NR	-	60	-	-	63.3
						18			-	68.8	-	-	52.1
		SP	48										
Madrid et al. (2011) [38]	Prospective cohort	SP	10	NR	12 months	13 years 5 months	NR	0	-	50	-	50	NR
		PMR	10						-	10	-	10	NR
Madrid et al. (2015) [30]	Prospective cohort	SP	37	2010–2011	at least 12 months		NR	0	-	94.5	-	81.1	NR
Mohamed et al. (2022) [39]	Prospective cohort	SP	20	2018–2020	1 year	4 years 11 months	NR	NR	0	15		15	80
Orr et al. (1987) [18]	Prospective cohort	PF	10	NR	~3 months	4–9 years	NR	0	90	20	90	20	NR
		PMR **	10			14–18 months	simultaneous primary repair		10	0	0	0	NR
Raymond et al. (2004) [40]	Prospective cohort	SP	17	NR	5.3 months (2.1–20)	14 years	NR	82.4	-	11.8	-	11.8	NR
Sullivan et al. (2010) [41]	Retrospective cohort	PF	104	1981–2008	3–6 months	8 year 7 months	NR	NR	-	8.65	-	1.92	75.9
Yamaguchi et al. (2016) [42]	Retrospective cohort	PMR	231	2007–2014	30.3 months (±20.5)	8 months	simultaneous primary repair	NR	-	0.87	-	0.43	47.6
Yamaguchi et al. (2016) [43]	Retrospective cohort	PF	38	2007–2014	38.2 months (±24.3)	7 years	NR	NR	-	23.7	-	2.63	81.6
Yamashita et al. (2008) [44]	Prospective cohort	PF	58	unclear	12 months	20 years	NR	0	-	36.2	-	-	NR
Ysunza et al. (2002) [45]	Randomized controlled trial	PF	25	1995–2000	at least 4 months	4 years 7 months	NR	NR	-	0	-	0	88
		SP	25			4 years 5 months			-	0	-	0	84
Zhao et al. (2021) [46]	Retrospective cohort	PF	52	2007–2017	6 years	8.9 years	Von Langenbeck, Furlow, two-flap	NR	-	31	-	31	NR
			30			23.3 years		NR	-	20	-	20	NR

PF: Pharyngeal Flap. SP: Sphincter Pharyngoplasty. PMR: Palatal Muscle Repositioning. NR or ‘–’: Not Reported or no specific period/follow-up time was reported on this outcome. ^a^: clinical signs of OSA: nasal obstruction, oral breathing, snoring, apneas, excessive daytime sleepiness, positive questionnaires (Epworth or Stanford Sleepiness Scale, STOP-BANG). ^b^: no hypernasality observed during speech assessment by speech therapist. *: bilateral buccinator myomucosal flaps. **: Intravelar velopalsty. ***: Sommerlad palatoplasty.

**Table 2:** A table that demonstrates the quality assessment including the risk of bias.

Study	Study Design	Level of Evidence (OCEBM 2011)	Reported OSA Outcomes (Symptoms/PSG)	PSG Monitoring	Reported Nasality Outcomes	Speech Assessment Tool	Speech Therapist	Blinded Speech Therapist	Post-Operative Assessment by Same Speech Therapist
Abdel-Aziz et al. (2008) [24]	Prospective case-series	4	Yes	Yes	Yes	Sell and Grunwell	Yes	No	No
Abdel-Aziz et al. (2011) [25]	Prospective case-series	4	Yes	Yes	Yes	Auditory Perceptual Assessment (APA)	Yes	Yes	No
Abdel-Aziz et al. (2018) [21]	Prospective case-series	4	Yes	Yes	No		Yes	No	No
Abyholm et al. (2004) [27]	Randomized controlled trial	2	Yes	Yes	Yes	four point scale	Yes	Yes	No
Boynuyoğun et al. (2023) [26]	Retrospective case-series	4	Yes	Yes	No		Yes	No	No
Campos et al. (2016) [28]	Prospective case-series	4	Yes	Yes	No		Yes	No	No
Chen et al. (1996) [29]	Retrospective case-series	4	Yes	No	Yes	In house assessment score	Yes	No	Yes
Denadai et al.(2017) [19]	Prospective case-series	4	Yes	No	Yes	Henningsson G, Universal parameters for reporting speech outcomes in individuals with cleft palate (Cleft palate craniofac Journal 2018)	Yes	Yes	Yes
El Anwar et al. (2017) [47]	Prospective case-series	4	Yes	No	Yes	Auditory Perceptual Assessment (APA)	Yes	Yes	Yes
Elsherbiny et al. (2020) [20]	Prospective case-series	4	Yes	No	Yes	In house assessment score	Yes	Yes	Yes
Emara et al. (2012) [31]	Prospective case-series	4	Yes	Yes	Yes	Auditory Perceptual Assessment (APA)	Yes	No	No
Hassib et al. (2005) [32]	Randomized controlled trial	2	Yes	Yes	Yes	unclear	Yes	No	No
Hsu et al. (2015) [33]	Retrospective case-series	4	Yes	Yes	Yes	In house assessment score	Yes	No	No
Junior et al. (2012) [34]	Retrospective case-series	4	Yes	Yes	Yes	In house assessment score	Yes	No	Yes
Liao et al. (2002) [22]	Prospective case-series	4	Yes	Yes	No	In house assessment score	Yes	No	Yes
Liao et al. (2003) [35]	Prospective case-series	4	Yes	Yes	No	In house assessment score	Yes	No	Yes
Lin et al. (1999) [23]	Retrospective case-series	4	Yes	Yes	Yes	Dalston and Warren 1985 + Mc Williams 1996	Yes	No	No
Liu et al. (2020) [36]	Retrospective case-series	4	Yes	Yes	Yes	the Speech Parameters Group 2008	Yes	No	Yes
Luo et al. (2019) [37]	Retrospective case-series	4	Yes	No	Yes	In house assessment score	Yes	No	Yes
Madrid et al. (2011) [38]	Prospective case-series	4	Yes	Yes	No	In house assessment score	Yes	No	Yes
Madrid et al. (2015) [30]	Prospective case-series	4	Yes	Yes	No	unclear	Yes	No	No
Mohamed et al. (2022) [39]	Prospective case-series	4	Yes	Yes	Yes	In house assessment score	Yes	No	Yes
Orr et al. (1987) [18]	Prospective case-series	4	Yes	Yes	No	unclear	No	No	No
Raymond et al. (2004) [40]	Prospective case-series	4	Yes	Yes	No	Borrel-Matsonnay classification	Yes	No	No
Sullivan et al. (2010) [41]	Retrospective case-series	4	Yes	Yes	Yes	Pittsburgh Weighted Values	Yes	No	No
Yamaguchi et al. (2016) [42]	Retrospective case-series	4	Yes	Yes	Yes	In house assessment score	Yes	No	Yes
Yamaguchi et al. (2016) (2) [43]	Retrospective case-series	4	Yes	Yes	Yes	In house assessment score	Yes	No	No
Yamashita et al. (2008) [44]	Prospective case-series	4	Yes	Yes	No	unclear	Yes	No	No
Ysunza et al. (2002) [45]	Randomized controlled trial	2	Yes	Yes	No	In house assessment score	Yes	Yes	Yes
Zhao et al. (2021) [46]	Retrospective case-series	4	Yes	Yes	No	unclear	Yes	No	Unclear

## 4. Discussion

This systematic review was conducted to determine the incidence of obstructive sleep apnea following secondary speech enhancing surgery in non-syndromic patients with a repaired cleft palate. Our data reveals a higher incidence of OSA (22%) after pharyngeal flap surgery and sphincter pharyngoplasty (23%) when compared to palatal muscle repositioning (3%). The choice for various surgical procedures for the treatment of cleft-related VPI is generally based on the pre-operative naso-endoscopic assessment of speech, the anatomy of the velopharyngeal airway, and the surgeon’s routine and preferences.

A high incidence of OSA in PF and SP procedures at both short- and long-term follow-ups was supported by the findings of a recent review and meta-analysis by Kurnik et al. [13].

The current data revealed more severe airway obstructive symptoms in PF than in both SP and PMR groups [21,22,23]. It is argued that PF may cause too much obturation, leading to a reduction in the cross-sectional area of the oropharynx and consequently leading to obstructive airway symptoms [23,48]. Additionally, the PF procedure enlarges the nasopharyngeal airway while the oropharyngeal airway narrows [49]. This compensatory constriction may potentially compromise the airway and lead to respiratory disorders (such as snoring and apnea) [48,49]. This is especially the case in children, while adults who undergo PF procedures are less severely affected due to the greater airway size, atrophied adenoids and tonsils, and a mature respiratory neuromuscular system [26,28].

During the SP procedure, the gap is primarily closed by a sphincter of two lateral ridges in the midline instead of the obturation method in the PF group [21,25,32]. This might explain the difference in the severity of the airway symptoms between these procedures.

The current data reveal a low incidence of OSA at short- and long-term follow-up and only mild and temporary symptoms of OSA in the PMR group. This finding is supported by reports from previous studies [22,30,50]. PMR is expected to have a less detrimental effect on the airway, as it is a dynamic procedure involving repositioning of the velar muscles into their common horizontal, more posterior, and anatomical orientation. This results in an increased velar length and enhancement of movement of the velum rather than obstructing the velopharyngeal space [35].

Contrary to the PMR group, the incidence of OSA in the PF and SP groups remained high in the long-term. Presentation of temporary symptoms of OSA shortly after surgery can possibly be attributed to the development of postoperative edema and swelling and can be the reason for the mild and temporary symptoms of OSA in the PMR group.

Data on speech resonance after surgical treatment of cleft-related VPI were extracted as secondary findings although this systematic search did not focus specifically on speech outcomes. Therefore selection bias should be taken into consideration when interpreting these outcomes.

The most rapid enhancement of speech was found in patients who underwent PF surgery, 82% of the subjects reached a normal speech resonance. Comparable results on speech were accomplished in the SP- and PMR groups, with 69% and 77% of the patients, respectively, achieving a competent velopharyngeal function.

Both findings on speech and airway obstructive problems could be explained by the higher degree of anatomical obstruction of the oropharynx that is caused using a pharyngeal flap compared to other surgical modalities that are discussed here.

In previous literature reviews on the outcomes of PMR, this procedure has been recommended as a preferred surgical intervention for the secondary treatment of VPI because of its effectiveness in enhancing speech and low risk of developing postoperative obstructive sleep apnea [13,51]. The rationale behind this approach is maximizing the palatal function before proceeding to the complete closure of the velopharyngeal gap with high-risk airway obstructive procedures such as pharyngoplasty. Although a reduction inf the need for pharyngoplasty with a higher risk of developing OSA is suggested to be significantly reduced when performing PMR, the indication for this procedure is considered mainly an abnormal orientation of the velar muscles and a small velopharyngeal gap size [51].

It is reported that different combinations between specific velopharyngeal closure patterns and gap size require a specific surgical approach for treating VPI [52]. Performing pharyngeal flap surgery on a patient with poor movement of the lateral pharyngeal wall is suggested to carry a higher chance of developing postoperative airway obstructive problems [52]. Therefore, visualizing the velopharyngeal anatomy, velopharyngeal gap size, and closure patterns by naso-endoscopic examination is a crucial step prior to the selection of surgical treatment for VPI.

### Limitations

This review has incorporated a systematic search through multiple databases. Although a large number of studies were found during the initial search, considerable heterogeneity was present between the included studies. This included the existence of various surgical modifications of the described techniques, the variability in patient characteristics such as age, demographics, extent of cleft, cleft surgical protocols, and the period of postoperative follow-up. This was of considerable concern when pooling and analyzing the results. Furthermore, the included studies did not report on additional factors that could have an effect on the anatomical and functional aspects of the velopharyngeal region. Among these are the frequency of cleft re-repair before secondary VPI repair, surgeon-specific modifications to surgical techniques, and airway competence. Conditions such as tonsillar and adenoid hypertrophy, obesity, and neuromuscular disorders that might also alter velopharyngeal function are reported inconsistently [53].

The pre-operative naso-endoscopy was not described in detail, and the reasons for choosing a particular treatment varied widely. Some studies provided an unclear or insufficient explanation to opt for certain procedures, such as ‘’the preference of the surgeon’’ or ‘’the protocol of the study’’. The quality of the included studies was limited. Most of the included studies scored level IV on the OCEBM evidence tool and also carried a high risk of selection bias due to allocation by clinicians. Only two randomized controlled trials were included.

The extraction and pooling of the data were performed in accordance with the Department of Statistics of our institution. As previously described, it is important to note that no statistical analyses were performed on the data. Therefore, no hard conclusions can be drawn from the pooling results.

Data on any signs and diagnosis of OSA were not reported consistently in the included studies. Only ten studies performed a preoperative evaluation to identify signs and diagnosis of OSA. The lack of preoperative evaluation of OSA may distort the outcomes and lead to bias when reporting the results.

The speech outcomes were not consistently reported using standardized criteria and assessment tools for an objective evaluation. Most studies used an ‘in-house assessment tool’ for the perceptual speech evaluation. Only six studies performed a speech assessment in which the observer was blinded to the patient’s characteristics and previous treatment.

It is evident that the lack of use of uniform methods to evaluate postoperative OSA and speech outcomes poses a challenge for the comparison of outcomes after various surgical treatments of cleft-related VPI. However, the comparison between the surgical techniques is crucial to improve the standard of care in patients with VPI while limiting the burden of airway obstruction.

## 5. Conclusions

The incidence of OSA after palatal muscle repositioning was 3% while reaching satisfactory results regarding speech outcome. Data suggest that while pharyngeal flap and sphincter pharyngoplasty are highly effective surgical modalities for the secondary treatment of VPI, they also carry a high risk of developing OSA with a reported incidence of 24% following each procedure. The choice of a surgical technique for the secondary treatment of VPI should be based on both naso-endoscopic examination and the pre-existent risk of the development of OSA. Further studies including standardized data collection on the pre- and post-operative existence of OSA and standardized PSG with additional speech assessment during long-term follow-up are imperative for an adequate comparison of airway obstructive outcomes and speech outcomes between different surgical approaches for the treatment of VPI to define best practice.

## Data Availability

The dataset can be requested by contacting the corresponding author, and the search strategy is provided in the supporting materials.

## Supplementary Materials

The following supporting information can be downloaded at: https://www.mdpi.com/article/10.3390/cmtr18010006/s1, Figure S1, Supplementary Digital Content. A figure that demonstrates the incidence of postoperative competent velopharyngeal function based on post-operative naso-endoscopy. Important note: NR (Not Reported) means that no data was available/or could be extracted from the included studies. Figure S2, Supplementary Digital Content. A figure that demonstrates the incidence of a closed velopharyngeal gap based on post-operative naso-endoscopy. NR (Not Reported) means that no data was available/or could be extracted from the included studies. Figure S3, Supplementary Digital Content. A figure that demonstrates the incidence of the absence of nasal air emission. NR (Not Reported) means that no data was available/or could be extracted from the included studies. Figure S4, Supplementary Digital Content. A figure that demonstrates the incidence of a normal intra-oral pressure. NR (Not Reported) means that no data was available/or could be extracted from the included studies. Figure S5, Supplementary Digital Content. A figure that demonstrates the incidence of normal speech intelligibility. NR (Not Reported) means that no data was available/or could be extracted from the included studies. Figure S6, Supplementary Digital Content. A figure that demonstrates the incidence of normal articulation. NR (Not Reported) means that no data was available/or could be extracted from the included studies. Figure S7, Supplementary Digital Content. A figure that shows the risk of bias using the Cochrane figures. Supplementary S1, Supplementary Digital Content. Overview of the search strategy across different databases.
