# Supplementary material for: Obstructive Sleep Apnea Following Secondary Velopharyngeal Insufficiency in Children with Non-Syndromic Cleft Palate: A Systematic Review
Source: Craniomaxillofac Trauma Reconstr. 2025 Jan 3;18(1):6. doi: 10.3390/cmtr18010006 (PMC11995821; doi:10.3390/cmtr18010006)
Supplement: Supplementary file 1 [file cmtr-18-00006-s001.zip › cmtr-3389710-supplementary.pdf]

Supplementary Digital Content:

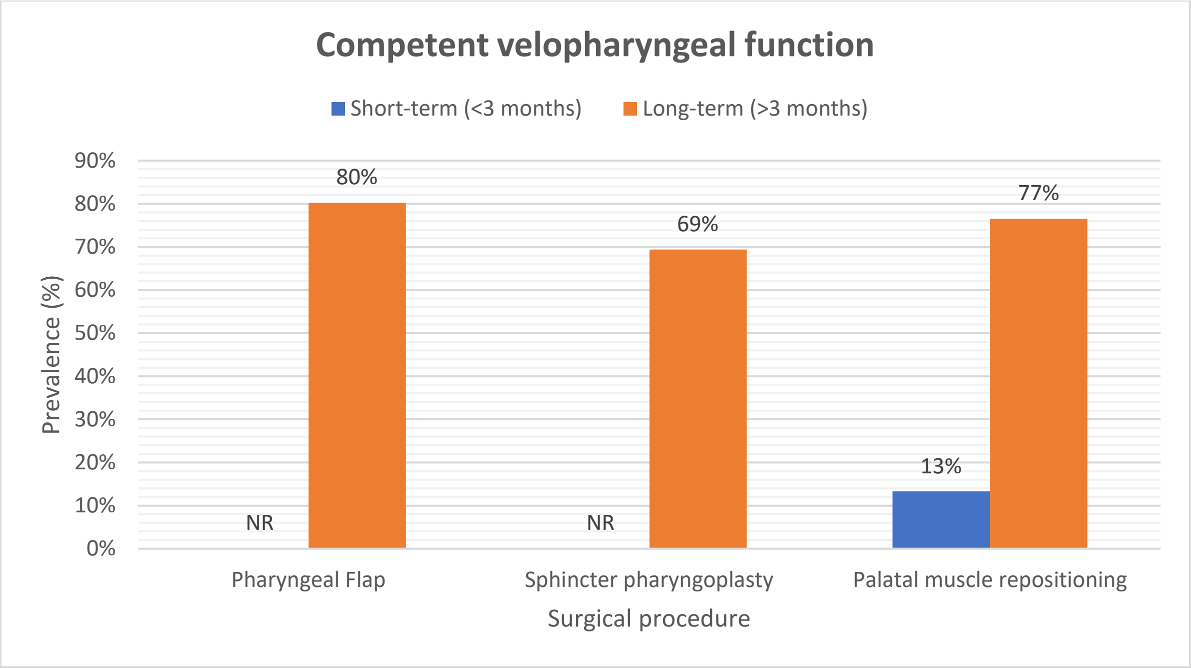

Figure S1 A figure that demonstrates the incidence of postoperative competent velopharyngeal function

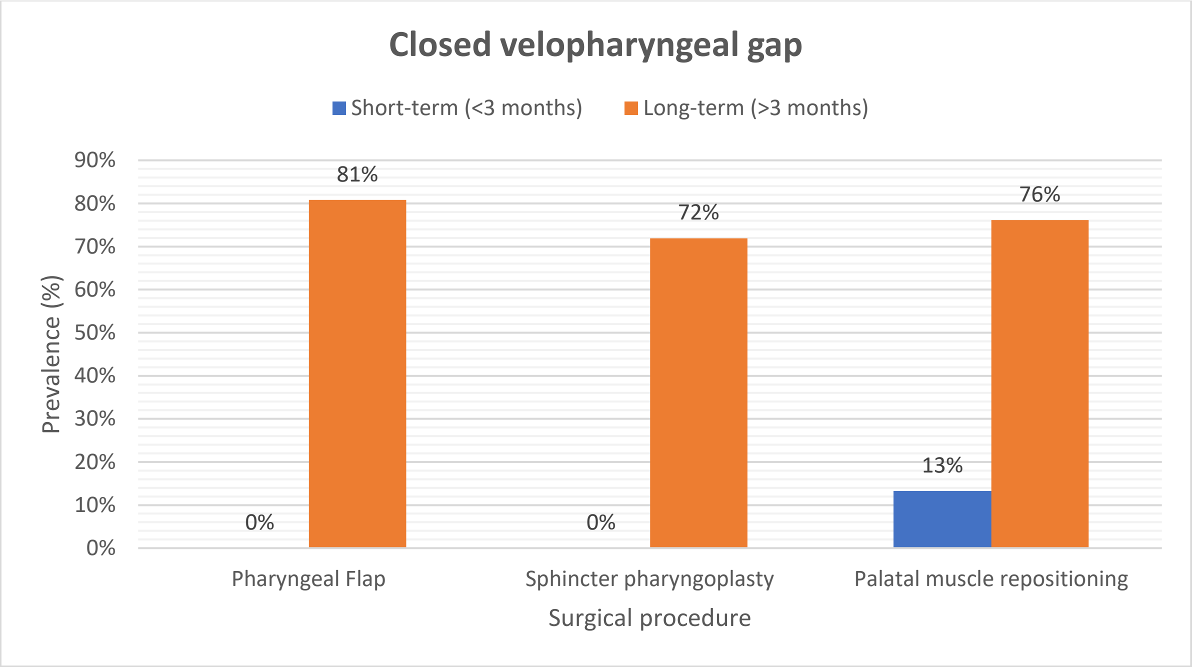

Figure S2 A figure that demonstrates the incidence of an adequate closure of the velopharyngeal gap based on post-operative naso-endoscopy

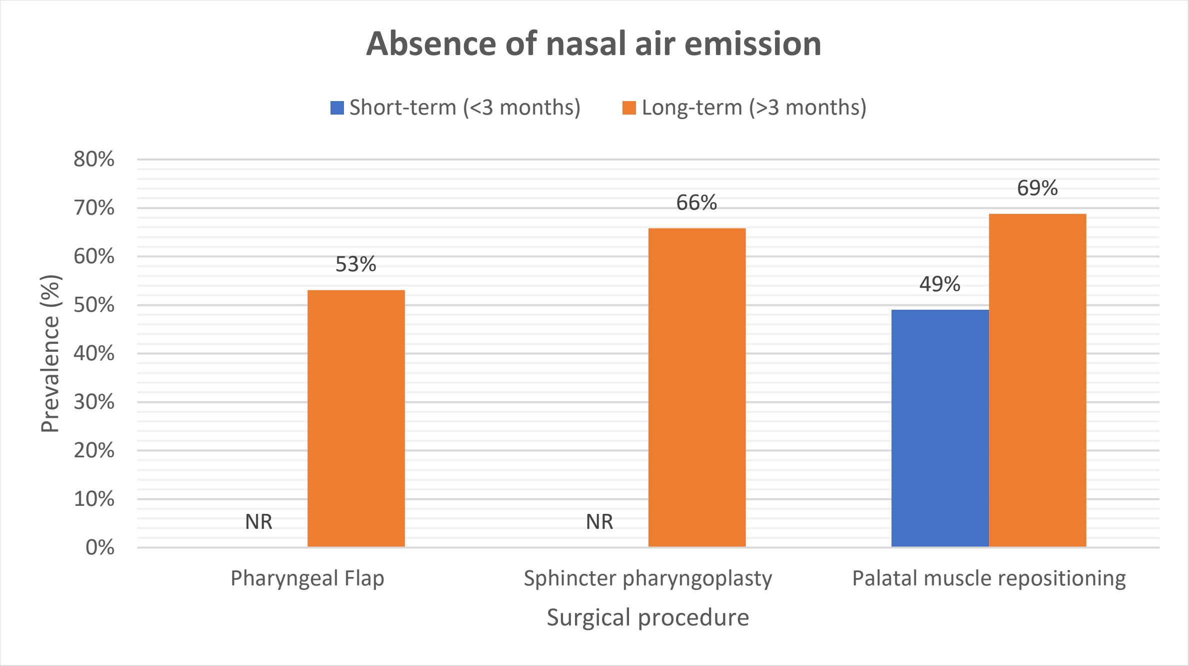

Figure S3 A figure that demonstrates the incidence of absence of nasal air emission post-operatively

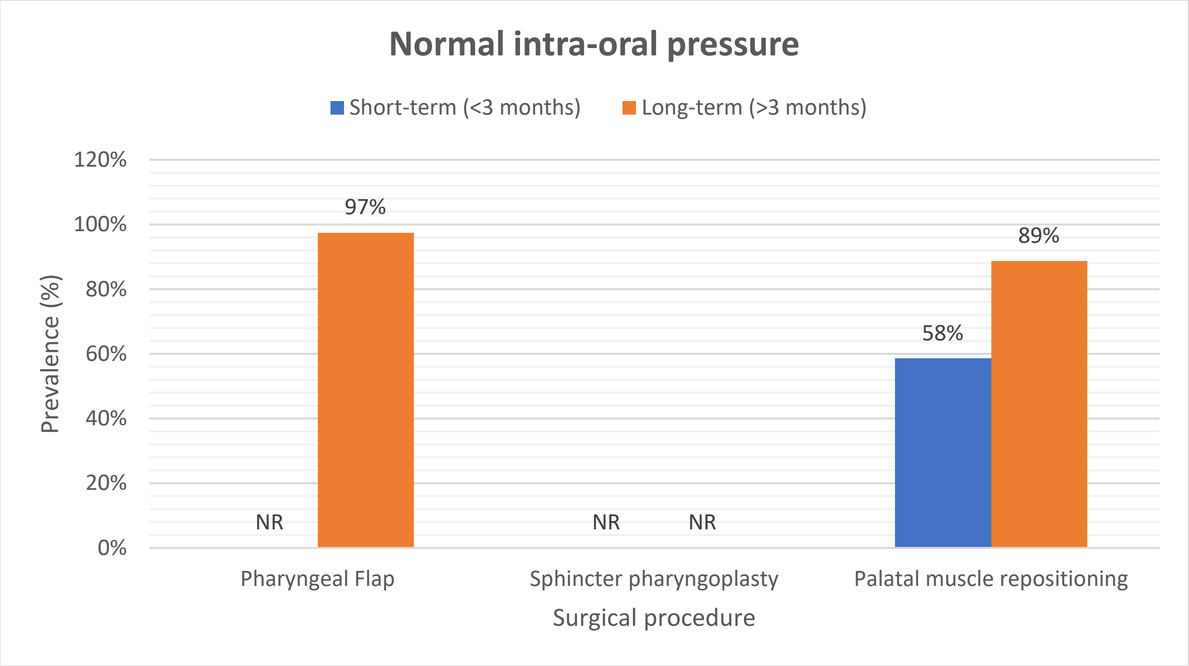

Figure S4 A figure that demonstrates the incidence of normal intra-oral pressure post-operatively

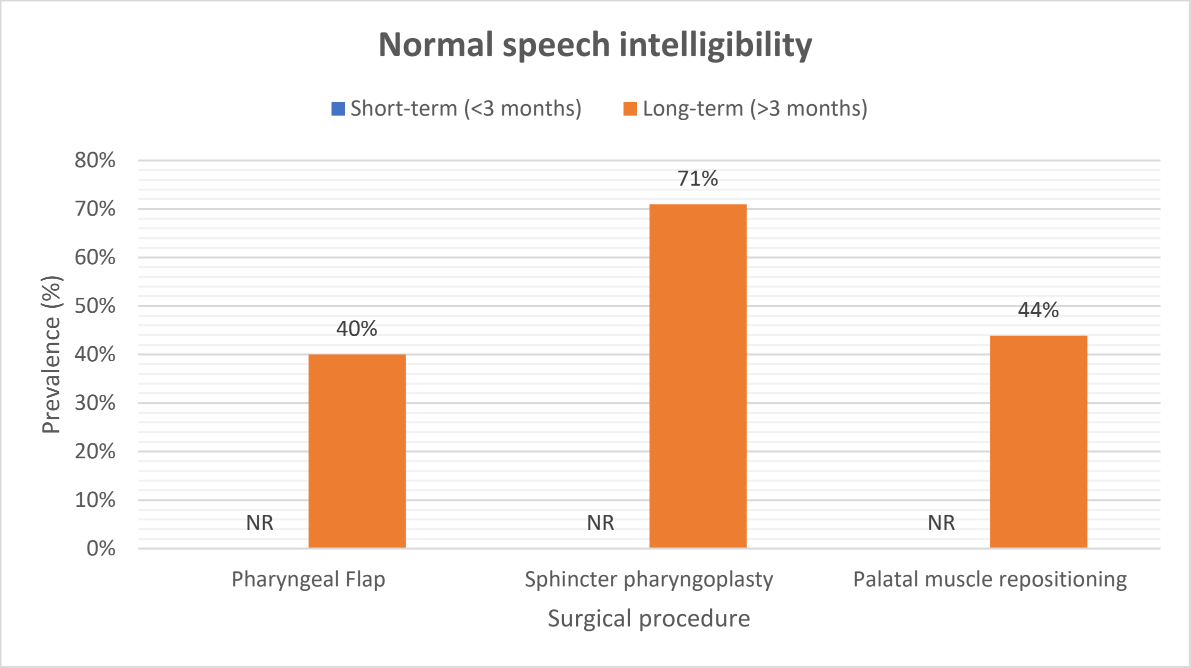

Figure S5 A figure that demonstrates the incidence of normal speech intelligibility post-operatively

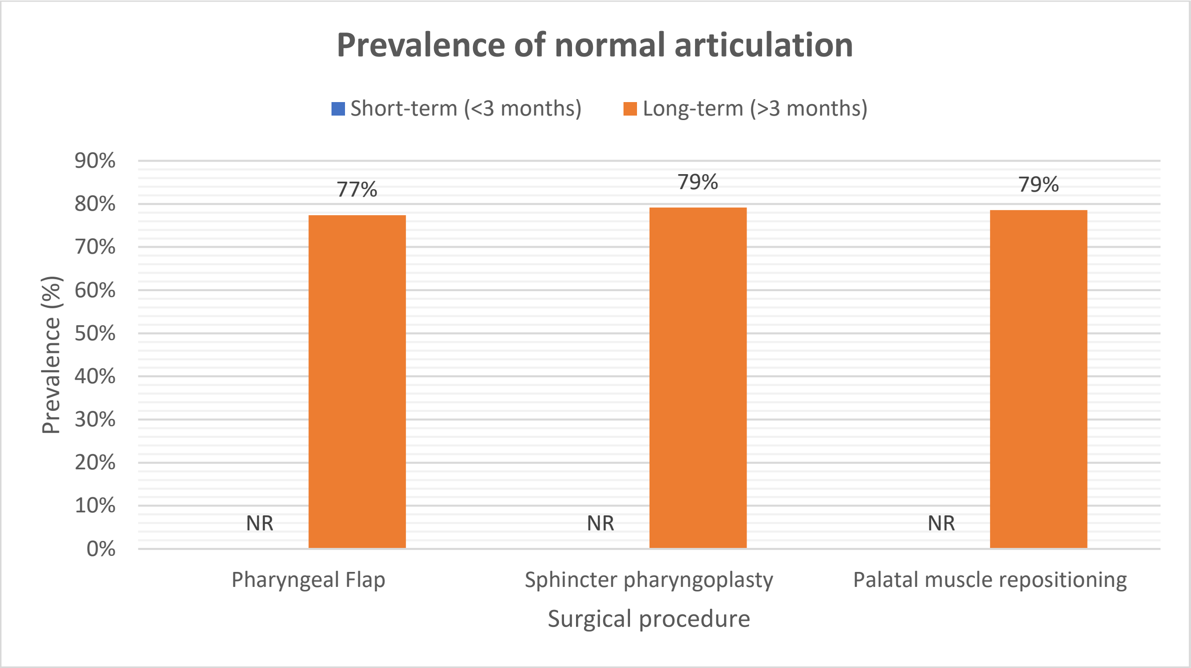

Figure S61 A figure that demonstrates the incidence of normal articulation post-operatively

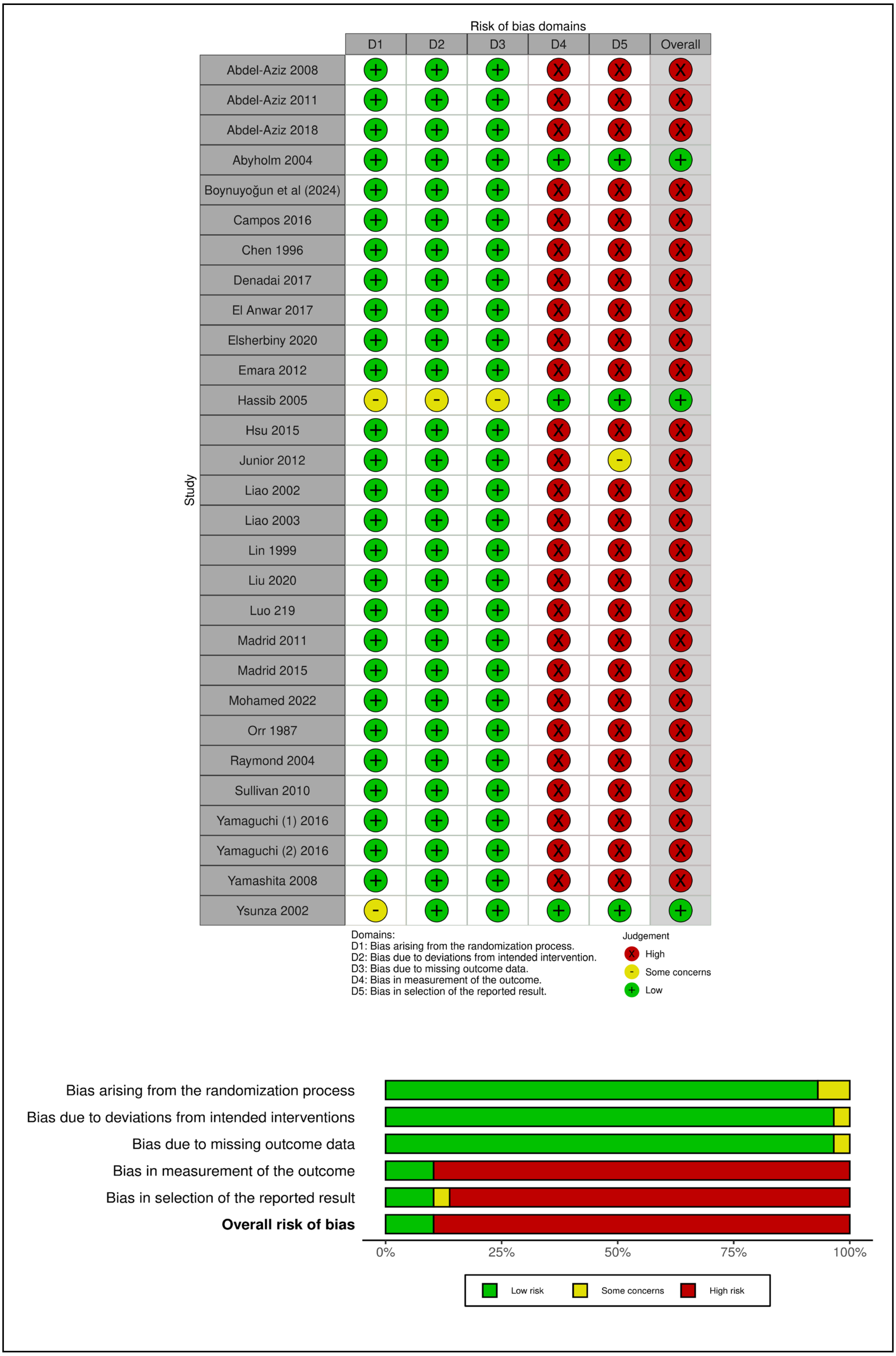

Figure S7 A figure that demonstrates the Risk of bias Cochrane figures

Supplementary S1: Overview of the search strategy across different databases.

embase.com
('pharynx reconstruction'/exp OR 'pharynx'/exp/dm_su OR 'palatopharyngeal incompetence'/exp OR 'adenoidectomy'/exp OR 'uvulopalatopharyngoplasty'/exp OR 'tonsillectomy'/exp OR (((pharyn* OR hypopharyn* OR nasopharyn* OR oropharyn* OR palatopharyn* OR velopharyn* OR adenoid* OR tonsil*) NEAR/3 (reconstruct* OR revision* OR repair* OR surg* OR incompeten* OR competen* OR failure* OR insufficien* OR sufficien* OR inadequa* OR deficien* OR dysfunction* OR function* OR augment* OR sling*)) OR pharyngoplast* OR velopharyngoplast* OR veloplast* OR palatopharyngoplast* OR uvulopalatopharyngoplast* OR adenoidectom* OR tonsillectom*):ab,ti) AND ('sleep disordered breathing'/exp OR apnea/exp OR 'polysomnography'/exp OR ((sleep NEAR/3 disorder* NEAR/3 breath*) OR apnea OR hypopnea OR apnoea OR hypopnoea OR osa OR osas OR osah OR osahs OR polysomnogra*):ab,ti) AND ('cleft palate'/exp OR 'cleft lip palate'/exp OR 'cleft lip'/de OR (cleft*):ab,ti)

Medline Ovid
(pharynx reconstruction/ OR Pharynx/su OR Velopharyngeal Insufficiency/ OR Adenoidectomy/ OR Tonsillectomy/ OR (((pharyn* OR hypopharyn* OR nasopharyn* OR oropharyn* OR palatopharyn* OR velopharyn* OR adenoid*) ADJ3 (reconstruct* OR revision* OR repair* OR surg* OR incompeten* OR competen* OR failure* OR insufficien* OR sufficien* OR inadequa* OR deficien* OR dysfunction* OR function* OR augment* OR sling*)) OR pharyngoplast* OR velopharyngoplast* OR veloplast* OR palatopharyngoplast* OR uvulopalatopharyngoplast* OR adenoidectom* OR tonsillectom*).ab,ti.) AND (exp apnea/ OR Polysomnography/ OR ((sleep ADJ3 disorder* ADJ3 breath*) OR apnea OR hypopnea OR apnoea OR hypopnoea OR osa OR osas OR osah OR osahs OR polysomnogra*).ab,ti.) AND (Cleft Palate/ OR cleft lip/ OR (cleft*).ab,ti.)

Cochrane CENTRAL
((((pharyn* OR hypopharyn* OR nasopharyn* OR oropharyn* OR palatopharyn* OR velopharyn* OR adenoid* OR tonsil*) NEAR/3 (reconstruct* OR revision* OR repair* OR surg* OR incompeten* OR competen* OR failure* OR insufficien* OR sufficien* OR inadequa* OR deficien* OR dysfunction* OR function* OR augment* OR sling*)) OR pharyngoplast* OR velopharyngoplast* OR veloplast* OR palatopharyngoplast* OR uvulopalatopharyngoplast* OR adenoidectom* OR tonsillectom*):ab,ti) AND (((sleep NEAR/3 disorder* NEAR/3 breath*) OR apnea OR hypopnea OR apnoea OR hypopnoea OR osa OR osas OR osah OR osahs OR polysomnogra*):ab,ti) AND ((cleft*):ab,ti)

Web of science
TS=(((((pharyn* OR hypopharyn* OR nasopharyn* OR oropharyn* OR palatopharyn* OR velopharyn* OR adenoid* OR tonsil*) NEAR/2 (reconstruct* OR revision* OR repair* OR surg* OR incompeten* OR competen* OR failure* OR insufficien* OR sufficien* OR inadequa* OR deficien* OR dysfunction* OR function* OR augment* OR sling*)) OR pharyngoplast* OR velopharyngoplast* OR veloplast* OR palatopharyngoplast* OR uvulopalatopharyngoplast* OR adenoidectom* OR tonsillectom*)) AND (((sleep NEAR/2 disorder* NEAR/2 breath*) OR apnea OR hypopnea OR apnoea OR hypopnoea OR osa OR osas OR osah OR osahs OR polysomnogra*)) AND ((cleft*)))

Google scholar

'pharynx|pharyngeal|velopharynx|velopharyngeal|adenoid|tonsil reconstruction|revision|repair|surgery|incompetence|insufficiency|deficiency'|pharyngoplasty| velopharyngoplasty|veloplasty 'sleep disordered breathing'|apnea|apnoea|polysomnography cleft "pharynx|pharyngeal|velopharynx|velopharyngeal|adenoid|tonsil reconstruction|revision|repair|surgery|incompetence|insufficiency|deficiency"|pharyngoplasty| velopharyngoplasty|veloplasty "sleep disordered breathing"|apnea|apnoea|polysomnography cleft
